# The first sexual associations in the genus *Darditilla* Casal, 1965 (Hymenoptera, Mutillidae)

**DOI:** 10.3897/zookeys.454.8558

**Published:** 2014-11-13

**Authors:** David R. Luz, Kevin A. Williams

**Affiliations:** 1 Laboratório de Biologia Comparada de Hymenoptera, Departamento de Zoologia, Universidade Federal do Paraná. Caixa Postal 19020, 81531-980 Curitiba, PR, Brazil; 2 Florida State Collection of Arthropods, Division of Plant Industry, Florida Department of Agriculture and Consumer Services, 1911 SW 34th St., Gainesville, FL 32608, USA

**Keywords:** Sphaeropthalminae, Sphaeropthalmini, Pseudomethocina, velvet ants, Neotropical, *formiga feiticeira*

## Abstract

New sex associations are proposed for four species of *Darditilla*: *Darditilla amabilis* (Gerstaecker, 1874); *Darditilla bejaranoi* Casal, 1968; *Darditilla debilis* (Gerstaecker, 1874); and *Darditilla felina* (Burmeister, 1854). *Darditilla botija* Casal, 1965, **syn. n.** is the male of *Darditilla amabilis*; the other three males were previously unknown. *Mutilla decorosa* Kohl, 1882, **syn. n.** is conspecific with *Darditilla felina*. Descriptions and extended diagnoses are provided for previously unknown males and for females that were not adequately described. These represent the first sex associations for the genus *Darditilla*.

## Introduction

The genus *Darditilla* Casal, 1965 was erected to include a single new species, *Darditilla botija* Casal, 1965, which was known from males only (Casal 1965). In his study, Casal suggested that six described females may belong to *Darditilla*, and specifically suggested that *Darditilla amabilis* (Gerstaecker, 1874) could be the female of *Darditilla botija*. In 1968, Casal described 28 new *Darditilla* species or subspecies, each known only from the female, and transferred seven described species into *Darditilla*. Subsequent authors have described new *Darditilla* species (Casal 1971, Fritz and Martinez 1974), transferred additional species into *Darditilla* (Nonveiller 1990), synonymized species or subspecies (Fritz and Martinez 1993, Quintero and Cambra 2001), and transferred species into *Pseudomethoca* Ashmead, 1899 (Quintero and Cambra 2001). Each of these subsequent publications has treated only females. As currently recognized, *Darditilla* includes 35 species known from females and one known from males.

Only seven described species of *Darditilla* occur in Brazil (Gerstaecker 1874, Nonveiller 1990, Quintero and Cambra 2001). For this study, two *Darditilla* sex associations, *Darditilla felina* (Burmeister, 1854) and *Darditilla bejaranoi* Casal, 1968, were made in southern and southeastern Brazil based on discovery of mating pairs. Two additional associations, *Darditilla amabilis* and *Darditilla debilis* (Gerstaecker, 1874), were then deduced based on overlapping ranges and morphological similarities. Only one of the males, *Darditilla botija* syn. n. of *Darditilla amabilis*, is known in the literature. Below, we present the first four sexual associations for the genus *Darditilla*, including the genotype. Extended diagnoses are provided for the three previously unknown males and each female studied here; the male of *Darditilla amabilis*, as *Darditilla botija* syn. n., was adequately described by Casal (1965). Photographs and pertinent figures are provided for each sex of each species studied herein.

## Material and methods

The following acronyms are used for institutions housing the material discussed in the current study:

AMNH American Museum of Natural History, New York, USA;

CASC Department of Entomology, California Academy of Sciences, San Francisco, California, USA;

DZUP Coleção de Entomologia Pe. Jesus Santiago Moure, Departamento de Zoologia da Universidade Federal do Paraná, Curitiba, Paraná, Brazil;

EMUS Department of Biology Insect Collection, Utah State University, Logan, Utah, USA;

MACN Museo Argentino de Ciencias Naturales, Buenos Aires, Argentina;

MLUH Martin Luther University Halle-Wittenberg, Halle, Germany;

MNRJ Museu Nacional, Universidade Federal do Rio de Janeiro, Rio de Janeiro, Brazil;

MZSP Museu de Zoologia da Universidade de São Paulo, São Paulo, Brazil;

NHM Natural History Museum, London, UK;

NMW Naturhistorisches Museum Wien, Vienna, Austria;

UFES Universidade Federal do Espírito Santo, Vitória, Espírito Santo, Brazil;

YPM Peabody Museum of Natural History, Yale University, New Haven, USA;

ZMB Museum für Naturkunde an der Universität Humboldt zu Berlin, Berlin, Germany.

We use the abbreviations T2, T3, etc., to denote the second, third, etc., metasomal terga while S2, S3, etc., denote the second, third, etc., metasomal sterna. To compare mesosomal length and width, the distance between the anteromedial pronotal margin (excluding the anterior collar) and the scutellar-scale apex is divided by the distance between the extreme posterolateral pronotal margins, the maximum mesosomal width. The digitus or cuspis length relative to the free paramere length is used here to quantify differences in genitalic structure. For ease of comparison and to facilitate identification without dissecting the genitalic capsule, the cuspis, digitus and paramere measurements are taken in dorsal view from the apical margin of the parapenial lobe to the apex of each respective structure. Using this method, all measurements can be taken from the dorsal view and a single anchor point can be used for all three measurements. These are not actual measurements of structure length, but an index to compare relative lengths; all provided length ratios of genitalic structures are based on these indices.

## Taxonomy

### Darditilla

Taxon classificationAnimaliaHymenopteraMutillidae

Casal, 1965

Darditilla Casal, 1965. Eos, Madrid 41: 9–18.

#### Type species.

*Darditilla botija* Casal, 1965, by original designation.

#### Diagnosis.

**Figure 1.**
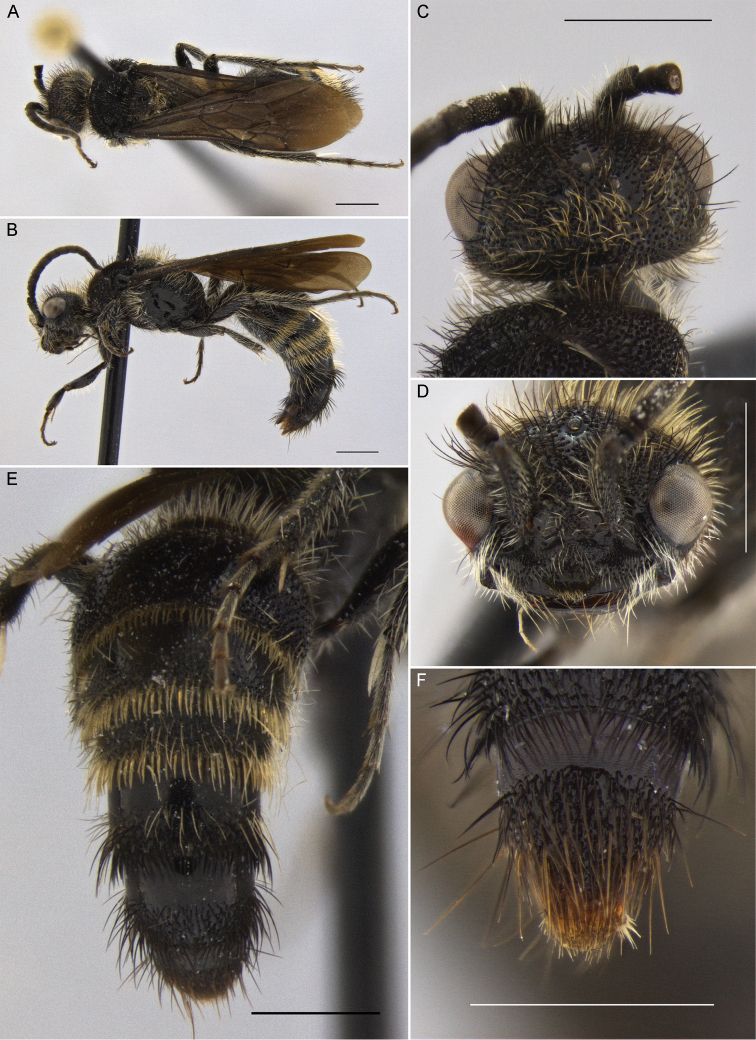
*Darditilla amabilis* (Gerstaecker), male: **A** habitus, dorsal view **B** habitus, lateral view **C** head, dorsal view **D** head, anterior view **E** metasoma, dorsal view **F** T5, T6 and pydigium, dorsal view. Scale bars: 1 mm.

**Male.** Males of *Darditilla* can be separated from other South American mutillid genera by the apical row of parallel bristles on T2–4 or T2–5 (e.g. Fig. 1E) and by the ventral margin of the clypeus that is preceded by a transverse furrow (e.g. Figs 3D, 7D) and is sometimes expanded into a broad fig-like structure over the mandibles (Fig. 1D). Additionally, *Darditilla* males have the scape bicarinate with a relatively flat or concave anterior surface between the carinae (although the dorsal carina is often obscure or obliterated); the axillae unarmed posteriorly; T1 broadly rounded into T2; the paramere downcurved apically; and the cuspis short and pad-like (e.g. Fig. 12).

**Female.** Females of *Darditilla* are most readily recognized by their granulate pygidium (e.g. Fig. 6E) and also have a unique combination of characters, wherein the clypeus is bidentate with the teeth slightly farther apart than the antennal tubercles (e.g. Fig. 8C); the mandible is acuminate apically and has its largest tooth situated in the basal half of the internal margin; the mesosoma is constricted anterior to the propodeal spiracles, lacks a scutellar scale, lacks a sharp dorsal tubercle directly anterior to the propodeal spiracle, and has the lateral mesonotal teeth small (e.g. Fig. 6A); T1 is broadly rounded into T2; and the metasomal setae are simple.

#### Species included.

There are 36 species in *Darditilla* (Nonveiller 1990, Quintero and Cambra 2001).

#### Distribution.

*Darditilla* species are known from throughout South America, putative members of *Darditilla* are known throughout Central America as well.

#### Remarks.

Casal (1965) described the genus from a single male specimen and used some synapomorphies of that species and its relatives in his generic description. The newly associated males described here match the diagnostic features listed by Casal and other authors in keys (e.g. Brothers 2006), but in two of the species: *Darditilla bejaranoi* and *Darditilla debilis*, the clypeus is less strongly modified. Rather than expanding forward to cover the mandibles, the ventral clypeal margin of these species is short, yet still has the ventral margin angled anteriorly.

*Darditilla* is apparently closely related to *Pseudomethoca* and could be nested within that genus. Males of some Nearctic and Central American *Pseudomethoca* species have thickened setae on T2–4 that resemble the bristles of *Darditilla* and some females currently placed in *Pseudomethoca* have a granulate pygidium. Further complicating this situation, Casal’s treatments of *Darditilla* focused on southern South America and the types of northern Neotropical *Pseudomethoca* species consistent with *Darditilla* were not available to him (Casal 1965, 1968a). Without phylogenetic analysis or careful study of both sexes of these species, we cannot determine which of these northern Neotropical *Pseudomethoca* should be transferred to *Darditilla*, or whether *Darditilla* is even a valid genus. We, therefore, maintain *Darditilla* using the aforementioned diagnoses and hope that this paper will facilitate the future studies needed to clarify the validity and limits of this genus.

### Darditilla amabilis

Taxon classificationAnimaliaHymenopteraMutillidae

(Gerstaecker, 1874)

Figs 1 –2 , 9 –12

Mutilla amabilis Gerstaecker, 1874. Arch. Naturgesch. 40: 63. Lectotype female, Brasilien, Alegrette, Sello S. (ZMB, examined), presently designated.
Mutilla braconina Burmeister, 1875. Bol. Acad. Nac. Sci. Cordoba 1: 488. Holotype female, (? MACN, not examined).
Darditilla botija Casal, 1965. Eos, Madrid 41: 14. Holotype male, República Argentina, Entre Ríos, Colón, II-1961, M. A. Zelich (AMNH, examined). **syn. n.**

#### Diagnosis.

**Male.** Males of *Darditilla amabilis* have a unique clypeus (Fig. 1D), which is widely transverse, almost covering the mandibles, with the ventral margin raised broadly and medially coming to an obtuse point with a subapical brush of golden setae, and have the penis valve unidentate apically (Fig. 11).

**Figure 2.**
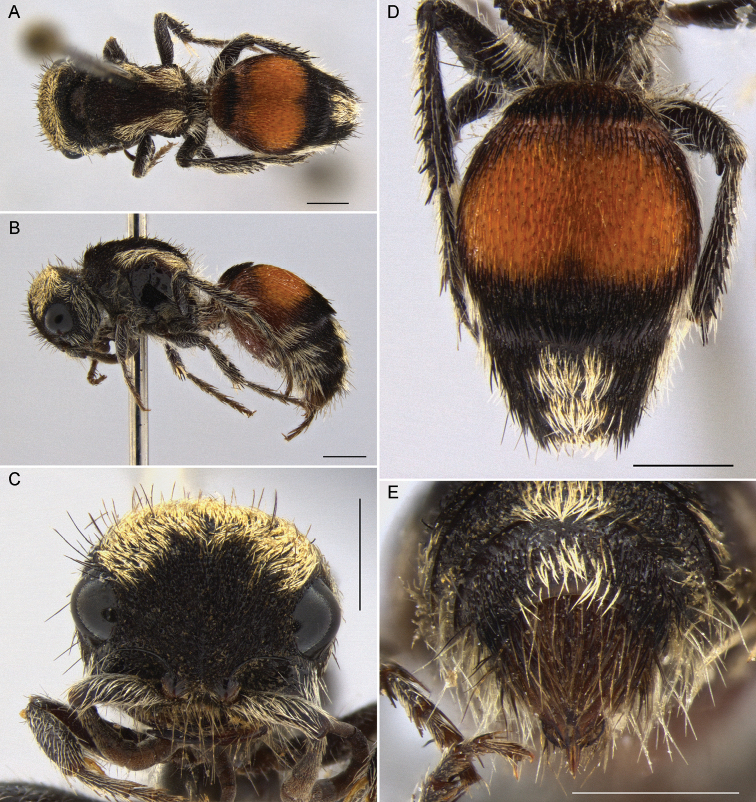
*Darditilla amabilis* (Gerstaecker), female: **A** habitus, dorsal view **B** habitus, lateral view **C** head, anterior view **D** metasoma, dorsal view **E** T4, T5 and pydigium, dorsal view. Scale bars: 1 mm.

**Female.** The female of *Darditilla amabilis* can be recognized by having T1, T2, S1 and S6 entirely orange (Fig. 2A), having an arcuate transverse band of recumbent pale golden setae on the vertex, and having a pair of longitudinal pale golden stripes in the mesosomal dorsum, which extend to the anterior margin of mesonotum (Fig. 2A).

#### Description.

**Male.** Body length 8.5 mm. The male was adequately described (as *Darditilla botija*) by Casal (1965).

**Extended female diagnosis.** Body length 7.6 mm. *Coloration.* Body and appendages reddish-black, except T1, T2, S1 and S6 entirely orange. Tibial spurs whitish. Vertex with dense, arcuate transverse band of recumbent pale golden setae, front and remainder of vertex with recumbent black setae; genal setae silver. Mesosomal dorsum covered with recumbent black setae, except laterally, with a pair of longitudinal pale golden stripes, extending to anterior margin of mesonotum. Posterior fringes of T1 and T2 black; T2 setae black anteriorly and posteriorly, pale golden mixed with black laterally, and reddish orange on orange integumental spots; T3–T6 clothed with black setae laterally and pale golden setae medially. *Head*. Transverse, posterior margin flat, occipital carina weak, but distinct. Head width 1.2 × pronotal width. Eye slightly ovate transversely, ommatidia distinct. Front, vertex and gena densely punctate. Genal carina well-defined, terminating in slightly sharp angle posterior to hypostomal carina. Clypeus with transverse glabrous concavity, margined by dorsal and ventral carinae, between widely separated lateral teeth. Mandible slender, tapering, bidentate apically (subapical tooth minute, distant from apex and usually obliterated through wear), unarmed ventrally. Antennal scrobe with complete dorsal carina. Antennal tubercle punctate basally, with weak scratches on anterior face, glabrous dorsally. Scape simple, moderately punctate. Flagellomere 1 1,7 × pedicel length; flagellomere 2 1.3 × pedicel length. *Mesosoma*. Mesosomal length 1.4 × width. Mesosomal dorsum coarsely reticulate, propodeal reticulae broader and shallower. Lateral pronotal carina extending to epaulet, humeral angle with moderately sharp obtuse angle. Mesopleuron densely punctate and setose, posterior margin defined by vertical carina. Metapleuron and lateral face of propodeum smooth and shining dorsally with isolated fine setae, micropunctate and densely setose ventrally. In dorsal view, mesosoma broadened to anterior third, strongly narrowed at propodeal spiracle, propodeum abruptly broadened. Scutellar scale lacking. Propodeum convex, dorsal and lateral faces not obviously differentiated. *Legs*. Foreleg with a few long strong articulated spines on posterior/lateral margins of tarsomeres. Mid- and hind tibiae each with one rows of prominent spines, 5 spines in each row; apical spurs finely serrated laterally. Hind tibia with distinct secretory pore on inner/posterior surface near base of inner spur. *Metasoma*. T1gradually broadened from base, not constricted apically, sessile with T2, 0.6 × as wide as T2; anterior face moderately punctate and setose. T2 densely punctate, punctures slightly smaller and sparser on orange spots; felt line broad, 0.5 × as long as T2 laterally. T3–5 densely punctate. Pygidium broad and slightly convex, lateral margins defined by distinct weakly bowed carina, posterior margin rounded and defined by indistinct carina, finely granulate. S1 punctate, with weak darkened median longitudinal carina. S2 moderately punctate. S3–5 densely punctate. S6 moderately punctate.

#### Material examined.

**Type material.** Holotype: *Darditilla botija*, ‘República Argentina\Entre Ríos\Colón\II-1961\M.A. Zelich’ (handwritten label) ‘HOLOTYPUS’ (red label) ‘Darditilla [male symbol]\botija\ Casal 1965 ’ (red label) [1 male: AMNH]; Lectotype: *Mutilla amabilis*, ‘Brasilien\Alegrette\Sello S.’ (handwritten label) ‘6611’ ‘Type’ (red label) [1 female: ZMB]. **Other material.** 2 males and 2 females as follows: ARGENTINA: Buenos Aires: Bolivar, I-60 (R.J. Llano) [1 female: DZUP]; BRAZIL: Rio Grande do Sul: Pelotas, 16m, 31°44'39"S, 52°13'22"W, 26.III.2004 (R.F. Krüger) [1 female: AMNH]; same locality, 16.IV.2004 (R.F. Krüger) [1 male: UFES]; Rio Grande, Taim, Mata do Nicola 32.5557°S, 52.5006W, 10–18.XII.2011, Arm. Malaise (Krüger & Kirst) [1 male: DZUP].

#### Distribution.

This species is widespread in Argentina and also occurs in Rio Grande do Sul, and Uruguay (Nonveiller 1990).

#### Host.

Unknown.

#### Remarks.

Gerstaecker (1874) described *Mutilla amabilis* based on two female specimens, one from Alegrete, Rio Grande do Sul State, Brazil and another from Paraná, Entre Ríos Province, Argentina. The specimen from Alegrete, deposited in the ZMB and bearing the labels cited above, was examined and is herein designated as a lectotype.

Casal (1965) hypothesized that *Darditilla botija* was the male of *Darditilla amabilis* based on geographical distribution in northern Argentina. Yet another overlapping distribution was found in Pelotas, Rio Grande do Sul State, Brazil. Only two other *Darditilla* females are known from Rio Grande do Sul: *Darditilla debilis* and *Darditilla infantilis* (Burmeister, 1875). The male of *Darditilla debilis* is described below; *Darditilla infantilis* is structurally similar to *Darditilla bejaranoi* and has a consistently small body size, precluding it from association with *Darditilla botija*. The newly described male of *Darditilla felina* has similar genitalia and clypeal modifications to *Darditilla botija*. Likewise, females of *Darditilla felina* are similar to those of *Darditilla amabilis* in the integumental markings of T2 and the genal carina. Distribution, similar body size, and morphological similarities to both sexes of *Darditilla felina* support the synonymy of *Darditilla botija* under *Darditilla amabilis*.

Burmeister (1875) had briefly described the supposed male of *Mutilla amabilis* from Paraná, Entre Ríos, Argentina. Later, André (1908) studied and redescribed the male that Burmeister (1875) originally associated with *Mutilla amabilis*; he pointed out that its identity and sexual association were doubtful. This male is clearly not conspecific with the male of *Darditilla amabilis* described above, most notably differing in its reddish basal metasomal segments. The description of this putative *Darditilla amabilis* male is, however, consistent with some Argentinean *Pseudomethoca* males.

### Darditilla bejaranoi

Taxon classificationAnimaliaHymenopteraMutillidae

Casal, 1968

Figs 3 –4 , 13 –16

Darditilla bejaranoi Casal, 1968. Rev. Soc. Ent. Arg. 30(1–4): 95. Holotype female, Brasil, Santa Catarina, Corupá, II-1953, A. Maller (AMNH, examined).

#### Diagnosis.

**Figure 3.**
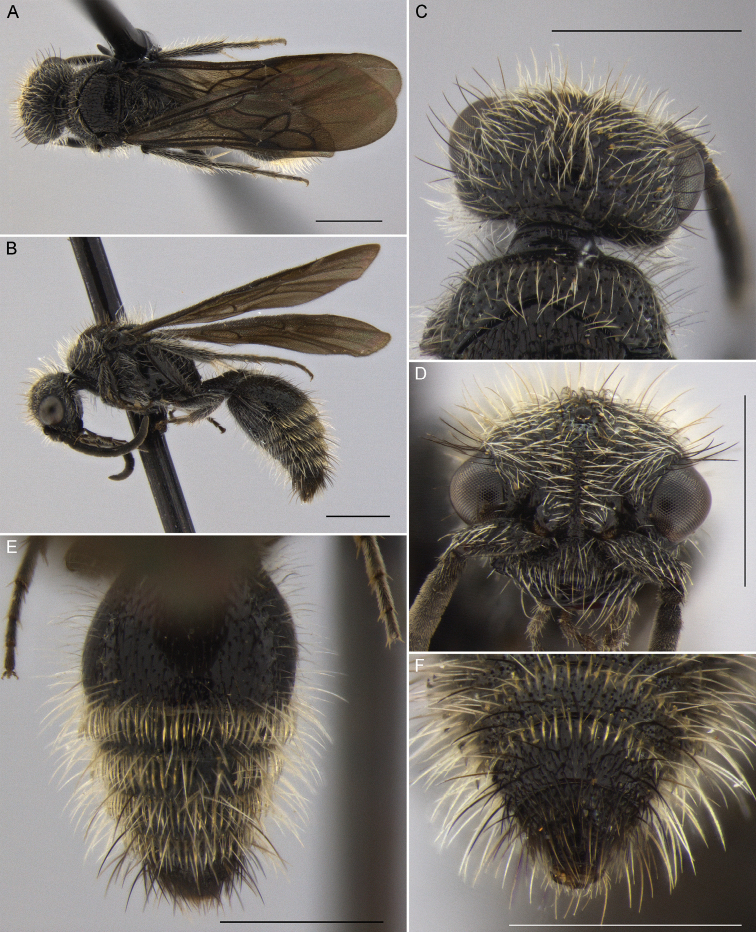
*Darditilla bejaranoi* Casal, male: **A** habitus, dorsal view **B** habitus, lateral view **C** head, dorsal view **D** head, anterior view **E** metasoma, dorsal view **F** T5, T6 and pydigium, dorsal view. Scale bars: 1 mm.

**Male.** The male of *Darditilla bejaranoi* can be recognized by having the ventral clypeal margin produced as a short transverse slightly upcurved impunctate lamella (Fig. 3D), by having the tegula truncate with a flat posterior face, and by having the bidentate penis valve teeth widely separated (Fig. 15).

**Figure 4.**
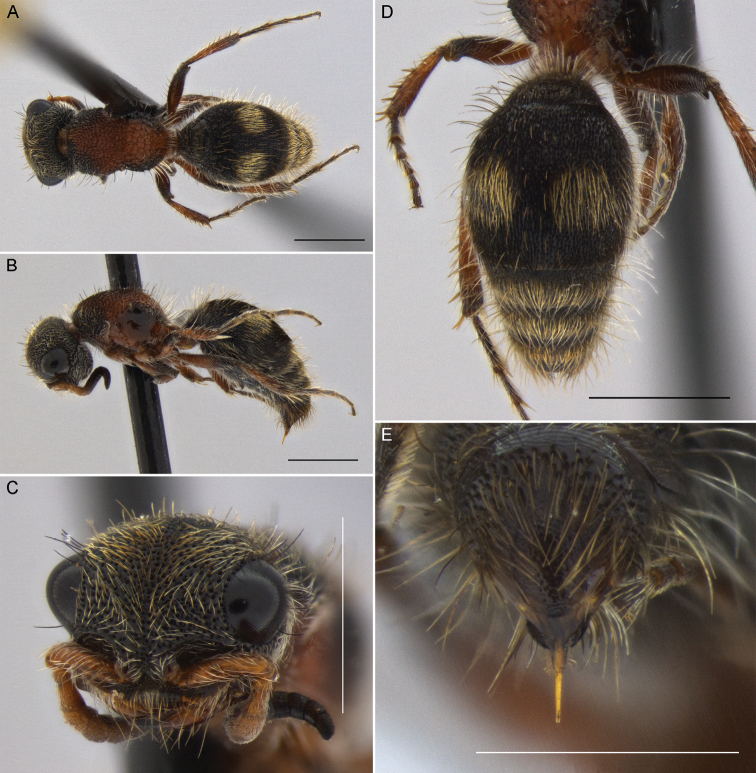
*Darditilla bejaranoi* Casal, female: **A** habitus, dorsal view **B** habitus, lateral view **C** head, anterior view **D** metasoma, dorsal view **E** T5 and pydigium, dorsal view. Scale bars: 1 mm.

**Female.** This female has a reddish mesosoma, with distinct black areas on the lateral pronotal dorsum and the posterior half of the pleurae (Fig. 4A, B), has lateral circular to transversely ovate silver setal spots on T2 (Fig. 4D), and has a strong hyaline median carina on S1.

#### Description.

**Male.** Body length 4.5–8 mm. *Coloration*. Body and appendages black, except mandibles and metasoma dark brown apically. Tibial spurs white. Forewing slightly and uniformly infuscated between veins, veins brown; hindwing slightly infuscated. Body clothed with whitish setae, except vertex, dorsomedial portion of pronotum, axilla, scutellum, disc of T2, T6 and T7 with scattered brownish setae, mesoscutum and tegula with dark brown setae, bristles of tergal fringes pale yellowish. *Head*. Rounded posteriorly. Head width 1.1 × pronotal width. Eye transversely ovate. Ocelli small; ocellocular distance 5.1 × length of lateral ocellus, interocellar distance 2.3 × lateral ocellus length. Occipital carina distinct, extending ventrally almost to level of oral fossa. Punctation on front dense, interspaces micropunctate; gena densely punctate, interspaces with sparse micropunctures; and vertex moderately punctate, interspaces glabrous. Gena ecarinate. Antennal scrobe broadly concave to eye margin, with transverse tubercle dorsally. Clypeus densely punctate, ventral margin produced as a short transverse slightly upcurved impunctate lamella. Flagellomere 1 1.2 × pedicel length; flagellomere 2 1.4 × pedicel length. Mandible convergent to bidentate apex, dorsal carina gradually becoming obsolete on inner tooth; edentate ventrally. *Mesosoma*. Epaulets weakly produced. Pronotal dorsum densely punctate; anterior face almost smooth; lateral face densely punctate. Tegula evenly convex anteriorly with abrupt vertical posterior face delimited by transverse dorsal carina, glabrous except with long recumbent setae anterolaterally and posteromesally. Mesoscutum with dense coarse punctures; posterolateral corner forming a small angulate lobe. Scutellum slightly convex, with coarse punctures. Axilla flat and punctate, except lateral margin with narrow vertical lamella. Metanotum surface obscured by dense shaggy mesally facing recumbent setae. Propodeum convex, broadly and deeply reticulate except smooth and shining adjacent to metapleuron. Mesopleuron moderately punctate, interspaces micropunctate. Metapleuron smooth and shining ventrally, setose and micropunctate dorsally. *Wings*. Forewing with moderate elongate sclerotized pterostigma; marginal cell broadly rounded and truncate apically; three submarginal cells, third scarcely delimited by obscure venation. *Legs*. Mid- and hind tibiae lacking strong spines, distinct apical secretory pore on inner surface near base of inner spur; spurs finely serrate on margins. *Metasoma*. T1gradually broadened from base, not constricted apically, sessile with T2, 0.6 × width of T2, sparsely punctate; apex with fringe of simple setae or thickened bristles. T2 with coarse to moderate punctures, interspaces smooth and generally broader than punctures; apex with fringe interspersed recumbent thickened parallel bristles; felt line 0.5 × lateral length of T2. T3–5 densely and finely punctate, covered with interspersed erect and recumbent setae, except fringes with row of bristles as in T2. T6 densely punctate, covered with less dense recumbent and erect setae. T7 in basal half with moderately spaced simple punctures and setae, interspaces glabrous; posterior half forming oval pygidium margined laterally and posteriorly by a strong sharp carina, apical margin rounded, surface flat, microgranulate with numerous large irregular transverse rugae. S1 punctate and setose, with medial longitudinal carina extending from base to apex. S2 moderately punctate. S3–6 moderately and finely punctate, with fairly sparse erect and recumbent setae. Lateral margins of S2–5 sometimes with similar bristles to those of T2–6. S7 transversely rectangular. Hypopygium smooth, moderately punctate, posteromedial margin with two approximate weak teeth medially. *Genitalia* (Figs 13–16). Paramere tapering apically, gradually curved ventrally, apices diverging slightly, dense setal brush on basoventral lateral margin, scattered setae along inner and lateral margins. Cuspis acute angulate apically, extending 0.2 × free length of paramere, with densely setose oblique ventral surface. Digitus laterally compressed and rounded apically, extending 0.2 × free length of paramere, asetose. Penis valve asetose, bidentate apically, basoventral margin expanded apically as blunt tooth, basodorsal margin with slight tubercle.

**Extended female diagnosis.** Body length 4.1–4.6 mm. *Coloration.* Head and metasoma black. Mesosoma reddish with variable blackish areas on lateral portion of pronotum and lateral face of mesosoma in posterior half. Appendages reddish, except mandible, flagellum, femora, and tibiae often darkened apically. Tibial spurs whitish. Front and vertex clothed with recumbent golden setae; genal setae silver. Mesosomal dorsum with sparse erect black setae, except often with silver setae laterally on pronotum and pale yellow setae dorsomedially on pronotum and posteriorly on propodeum. Posterior fringes of T1 and T2 black; T2 with lateral circular to transversely ovate silver setal spots; T3–6 clothed with silver setae. *Head*. Transverse, posterior margin flat, occipital carina obscure. Head width 1.4 × pronotal width. Eye slightly ovate transversely, ommatidia distinct. Front and vertex densely punctate; gena moderately punctate. Genal carina well-defined, extending nearly to hypostomal carina. Clypeus with shallow transverse glabrous concavity, margined by dorsal and ventral carinae, between widely separated lateral teeth. Mandible slender, tapering, bidentate apically (subapical tooth minute, distant from apex and usually obliterated through wear), unarmed ventrally. Antennal scrobe with complete dorsal carina. Antennal tubercle punctate basally and laterally. Scape simple, moderately punctate. Flagellomere 1 1.5 × pedicel length; flagellomere 2 1.4 × pedicel length. *Mesosoma*. Mesosomal length 1.8 × width. Mesosomal dorsum coarsely reticulate, propodeal reticulae broader and shallower. Lateral pronotal carina extending to epaulet, humeral angle shallowly obtusely angulate. Mesopleuron densely punctate and setose, posterior margin defined by vertical carina. Metapleuron and lateral face of propodeum smooth and shining dorsally with isolated fine setae, micropunctate and densely setose ventrally. In dorsal view, mesosoma broadened to anterior third, strongly narrowed at propodeal spiracle, propodeum abruptly broadened. Scutellar scale lacking. Propodeum convex, dorsal and lateral faces not obviously differentiated. *Legs*. Foreleg with a few long strong articulated spines on posterior/lateral margins of tarsomeres. Mid- and hind tibiae each with two rows of prominent spines, 2–4 spines in each row; apical spurs finely serrated laterally. Hind tibia with distinct secretory pore on inner/posterior surface near base of inner spur. *Metasoma*. T1gradually broadened from base, not constricted apically, sessile with T2, 0.5 × as wide as T2; anterior face moderately punctate and setose. T2 densely punctate and setose, punctures slightly larger and sparser anterolaterally; felt line broad, 0.4 × as long as T2 laterally. T3–5 densely punctate. Pygidium broad and slightly convex, lateral margins defined by distinct weakly bowed carina, posterior margin rounded and defined by indistinct carina, granulate, posterior granulae often more sparse, anterior granulae often merging to obscure striae or rugae. S1 punctate, with strong hyaline median carina. S2 moderately punctate. S3–5 densely punctate. S6 moderately punctate.

#### Material examined.

**Type material.** Holotype: ‘Brasil\Santa Catarina\Corupá\II-1953\A. Maller’ (handwritten label) ‘HOLOTYPUS’ (red label) ‘Darditilla [female symbol]\bejaranoi\ Casal 1968’ (red label) [1 female: AMNH]. **Other material.** 18 males and 49 females as follows: ARGENTINA: Corrientes: Ytuzaingo, III.1982 (M.A. Fritz) [1 female, 1 male: AMNH]; Entre Ríos: Colón, Parque Nacional, X.1974 (M.A. Fritz) [1 female: AMNH]; same locality, III.1982 (M.A. Fritz) [1 female: AMNH]; same locality, date unknown (M.A. Fritz) [1 female: AMNH]; same locality, XII.1973 (M.A. Fritz) [1 male: AMNH]; same locality, I.1974 (M.A. Fritz) [1 male: AMNH]; BRAZIL: Minas Gerais: Belo Horizonte, Museu de História Natural, 22.III.1998 (G.A.R. Melo) [1 female: DZUP]; 16 km S de Berizal, Serra do Anastácio, 18.XII.2012 (G.A.R. Melo) [1 male: DZUP]; 7 km S Itanhandu, 14.XI.2005 (L.R.R. Faria Jr.) [1 female: DZUP]; near Timoteo, 1–14.II.1999 (E.R. DePaula) [1 female: EMUS]; Paraná: Piraquara, Mananciais da Serra, 27.III.2003 (E.Q. Garcia) [1 female: DZUP]; Ponta Grossa, Parque Estadual da Vila Velha, 23.XI.2001 (G.A.R. Melo) [1 female: DZUP]; same locality, 15.XI.2003 (G.A.R. Melo & R.B. Gonçalves) [1 female: DZUP]; same locality, 5.IV.2013 (K.A. Williams) [10 females: DZUP]; same locality, 6.IV.2013 (K.A. Williams) [11 females: DZUP]; Rio de Janeiro: Mendes, 23.IV.1936 (Borgmeier) [1 pair *in copula*: MNRJ]; Petrópolis, Alto da Serra, 18.II.1962 (H. Cesar) [1 male: MNRJ]; km 47, estrada Rio-São Paulo, 24.X.1944 (Wygodzinsky) [1 female: MNRJ]; Santa Catarina: Corupa, various dates [5 females: MNRJ]; Galheta, P. Mole, 5.X.1988 (C.R.F. Brandão) [1 female: MZSP]; São Paulo: Barueri, V.1958 (K. Lenko) [1 female: MNRJ]; Botucatu, Cerrado, Armadilha Solo, 19.III.1987 (L.C. Forti & I.M.P. Rinaldi) [1 female: MZSP]; Cajuru, Fazenda Rio Grande, 18.XII.1999–10.I.2000 (G.A.R. Melo & Nascimento) [2 males: DZUP]; same locality, 10.I–1.II.2000 (G.A.R. Melo & Nascimento) [3 males: DZUP]; same city, Fazenda Santa Carlota, 17.XI-2.XII.1999 (G.A.R. Melo & Nascimento) [4 males: DZUP]; Campo Limpo, 20.II.1966 (W.W. Kempf) [2 females: MZSP]; Caraguata, Reserva Florestal, 40m, II.1963 (F. Werner, U. Martins, & L. Silva) [1 female: MZSP]; Cosmópolis, 22.I.1974 (J.G. Rozen et al.) [2 females: AMNH]; Ribeirão Grande, P.E. Intervales, ponto 5, 24°16'23"S, 48°25'22"W, 22.XII.2009 (N.W. Perioto) [1 male: MZSP]; Ribeirão Preto, Campus da USP, 12.XI.1998 (G.A.R. Melo) [1 female: DZUP]; Rio Claro, Floresta Estadual Edmundo Navarro de Andrade, 10.IX–1.X.2005 (A.P. Aguiar & J.T. Dias) [1 male: MZSP]; São Jose dos Campos, 8–14.III.1999 (E.R. DePaula) [1 female: EMUS]; same locality, 22–29.IX.1999 [1 female: EMUS]; São Paulo, 10.XI.1972 (G.E. Bohart) [1 male: EMUS].

#### Distribution.

This species is known from the Atlantic Rainforest of Brazil and surrounding areas of Argentina.

#### Host.

Unknown.

#### Remarks.

The sexes of *Darditilla bejaranoi* are associated based on a mating pair found in the MNRJ. Additionally, M.A. Fritz collected males and females in the same locality twice in Argentina. The male and female are similar in size and geographical distribution.

Females vary in mesosomal coloration, with some specimens having only a small dark brown patch on the pronotal dorsum and others having the mesosomal dorsum and pleurae extensively darkened. The setal spots of T2 also vary slightly in shape, from perfectly circular to transversely ovate. In Casal’s key (1968a) specimens with transversely ovate setal spots will run to couplet 25, where they can be separated from *Darditilla bachmanni* Casal, 1968 and *Darditilla nelidae* Casal, 1968 by the entirely black fringe of T1 (T1 with extensive white setae in *Darditilla bachmanni* and *Darditilla nelidae*) and geographical distribution (*Darditilla bachmanni* from Bolivian Amazon and *Darditilla nelidae* from central Argentina).

### Darditilla debilis

Taxon classificationAnimaliaHymenopteraMutillidae

(Gerstaecker, 1874)

Figs 5 –6 , 17 –20

Mutilla debilis Gerstaecker, 1874. Arch. Naturgesch. 40: 60. Holotype female, Brasil. mer, Sello. (ZMB, examined).

#### Diagnosis.

**Figure 5.**
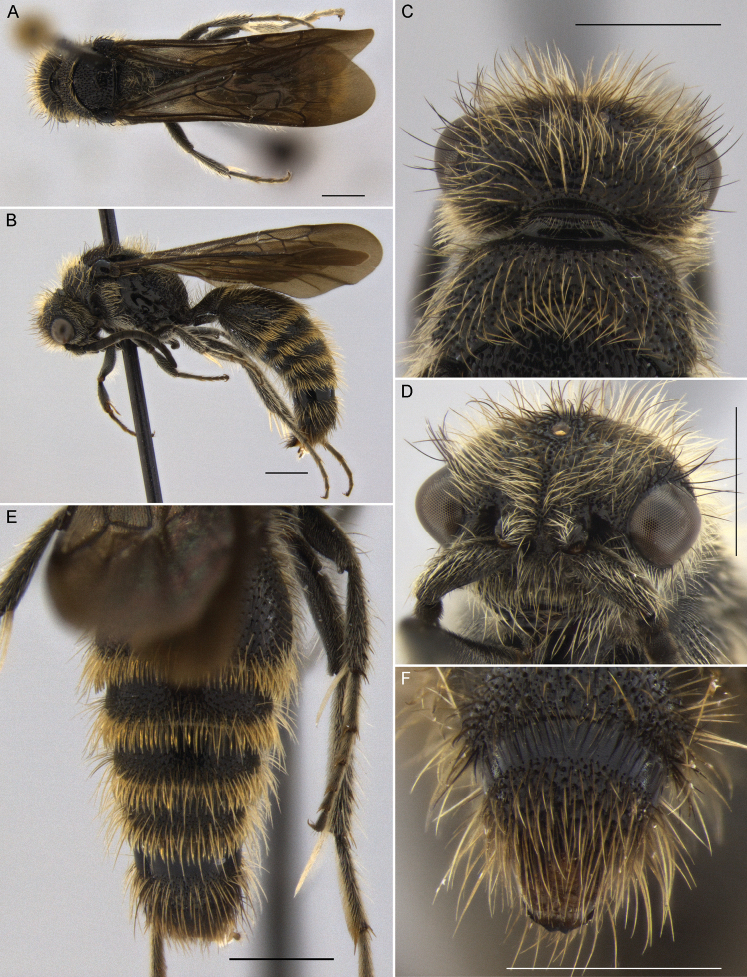
*Darditilla debilis* (Gerstaecker), male: **A** habitus, dorsal view **B** habitus, lateral view **C** head, dorsal view **D** head, anterior view **E** metasoma, dorsal view **F** T5, T6 and pydigium, dorsal view. Scale bars: 1 mm.

**Male.** Males are similar to *Darditilla bejaranoi*, but have a simply convex tegula, have the ventral impuctate lamella of the clypeus less produced than the preceding species (Fig. 5D), and have the apical and preapical teeth of the penis valve coalescent (Fig. 19).

**Figure 6.**
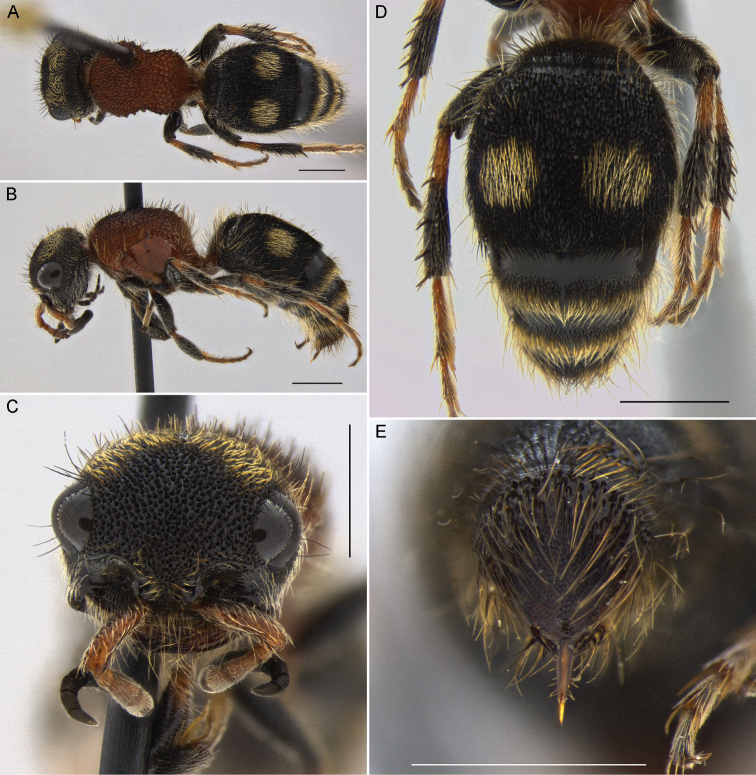
*Darditilla debilis* (Gerstaecker), female: **A** habitus, dorsal view **B** habitus, lateral view **C** head, anterior view **D** metasoma, dorsal view **E** T5 and pydigium, dorsal view. Scale bars: 1 mm.

**Female.** The female of *Darditilla debilis* is similar to *Darditilla bejaranoi*, but can be recognized by its reddish mesosoma, at most having reduced dark stains laterally (Fig. 6A); by its pygidium, which is densely granulate throughout (Fig. 6E); and by its S1, which has a weak darkened median longitudinal carina.

#### Description.

**Male.** Body length 5.7–8.4 mm. *Coloration*. Body and appendages black, except mandibles and metasoma dark brown apically. Tibial spurs white. Forewing slightly and uniformly infuscated between veins, veins brown; hindwing slightly infuscated. Body clothed with whitish setae, except disc of T2 and T7 with scattered brownish setae, mesoscutum, tegula, and T6 with dark brown setae, bristles of tergal fringes silver to pale yellowish. *Head*. Rounded posteriorly. Head width 1.0 × pronotal width. Eye transversely ovate. Ocelli small; ocellocular distance 7.1 × length of lateral ocellus, interocellar distance 3.1 × lateral ocellus length. Occipital carina distinct, extending ventrally almost to level of oral fossa. Punctation on front dense, interspaces micropunctate; gena densely punctate, interspaces with sparse micropunctures; and vertex moderately punctate, interspaces glabrous. Gena ecarinate. Antennal scrobe broadly concave to eye margin, with transverse tubercle dorsally. Clypeus densely punctate, ventral margin produced as a short slightly upcurved transverse impunctate lamella. Flagellomere 1 1.4 × pedicel length; flagellomere 2 1.6 × pedicel length. Mandible convergent to bidentate apex, dorsal carina gradually becoming obsolete on inner tooth; edentate ventrally. *Mesosoma*. Epaulets weakly produced. Pronotal dorsum densely punctate; anterior face almost smooth; lateral face densely punctate. Tegula evenly convex, glabrous except with long recumbent setae anterolaterally and posteromesally. Mesoscutum with dense coarse punctures; posterolateral corner forming a small angulate lobe. Scutellum slightly convex, with coarse punctures. Axilla flat and punctate, except lateral margin with narrow vertical lamella. Metanotum surface obscured by dense shaggy mesally facing recumbent setae. Propodeum convex, broadly and deeply reticulate except smooth and shining adjacent to metapleuron. Mesopleuron moderately punctate, interspaces micropunctate. Metapleuron smooth and shining ventrally, setose and micropunctate dorsally. *Wings*. Forewing with moderate elongate sclerotized pterostigma; marginal cell broadly rounded and truncate apically; three submarginal cells, third scarcely delimited by obscure venation. *Legs*. Mid- and hind tibiae lacking strong spines, distinct apical secretory pore on inner surface near base of inner spur; spurs finely serrate on margins. *Metasoma*. T1 gradually broadened from base, not constricted apically, sessile with T2, 0.6 × width of T2, sparsely punctate; apex with fringe of simple setae or thickened bristles. T2 with coarse to moderate punctures, interspaces smooth and generally broader than punctures; apex with fringe interspersed recumbent thickened parallel bristles; felt line 0.5 × lateral length of T2. T3–5 densely and finely punctate, covered with interspersed erect and recumbent setae, except fringes with row of bristles as in T2. T6 densely punctate, covered with less dense recumbent and erect setae. T7 coarsely and densely punctate, punctures uneven medially, appearing rugose; posterior half forming oval pygidium margined laterally and posteriorly by a strong sharp carina, apical margin rounded, surface flat, microgranulate with numerous large irregular transverse rugae. S1 punctate and setose, with medial longitudinal carina extending from base to apex. S2 moderately punctate. S3–6 moderately and finely punctate, with fairly sparse erect and recumbent setae. Lateral margins of S2–5 sometimes with similar bristles to those of T2–6. S7 transversely rectangular. Hypopygium coarsely punctate, posteromedial margin with two approximate weak teeth medially. *Genitalia* (Figs 17–20). Paramere tapering apically, slightly curved ventrally on basal half, dense setal brush on basoventral lateral margin, scattered setae along inner and lateral margins. Cuspis acute angulate apically, extending ~0.3 × free length of paramere, with densely setose oblique ventral surface. Digitus laterally compressed and rounded apically, extending ~0.3 × free length of paramere, asetose. Penis valve asetose, bidentate apically with apical and preapical tooth, basoventral margin slightly expanded apically as blunt tooth, basodorsal margin with slight tubercle.

**Extended female diagnosis.** Body length 4.8–10 mm. *Coloration.* Head and metasoma black. Mesosoma reddish, rarely with dark stains laterally. Appendages blackish, often reddish basally. Tibial spurs whitish. Vertex with arcuate transverse band of recumbent silver to golden setae, front and remainder of vertex with recumbent black setae; genal setae silver. Mesosomal dorsum with sparse erect black setae, except anterior margin pronotum with pale yellow setae. Posterior fringes of T1 and T2 black; T2 with lateral circular silver setal spots; T3–6 clothed with black setae basally and silver setae apically. *Head*. Transverse, posterior margin flat, occipital carina obscure. Head width 1.3 × pronotal width. Eye slightly ovate transversely, ommatidia distinct. Front and vertex densely punctate; gena moderately punctate. Genal carina well-defined, extending nearly to hypostomal carina. Clypeus with transverse glabrous concavity, margined by dorsal and ventral carinae, between widely separated lateral teeth. Mandible slender, tapering, bidentate apically (subapical tooth minute, distant from apex and usually obliterated through wear), unarmed ventrally. Antennal scrobe with complete dorsal carina. Antennal tubercle punctate basally, with weak scratches on anterior face, glabrous dorsally. Scape simple, moderately punctate. Flagellomere 1 1.9 × pedicel length; flagellomere 2 1.8 × pedicel length. *Mesosoma*. Mesosomal length 1.3 × width. Mesosomal dorsum coarsely reticulate, propodeal reticulae broader and shallower. Lateral pronotal carina extending to epaulet, humeral angle with sharp obtuse angle. Mesopleuron finely densely punctate and setose, posterior margin defined by vertical carina. Metapleuron and lateral face of propodeum smooth and shining dorsally with isolated fine setae, micropunctate and densely setose ventrally. In dorsal view, mesosoma broadened to anterior third, strongly narrowed at propodeal spiracle, propodeum abruptly broadened. Scutellar scale lacking. Propodeum convex, dorsal and lateral faces not obviously differentiated. *Legs*. Foreleg with a few long strong articulated spines on posterior/lateral margins of tarsomeres. Mid- and hind tibiae each with two rows of prominent spines, 2–4 spines in each row; apical spurs finely serrated laterally. Hind tibia with distinct secretory pore on inner/posterior surface near base of inner spur. *Metasoma*. T1gradually broadened from base, not constricted apically, sessile with T2, 0.6 × as wide as T2; anterior face moderately punctate and setose. T2 densely punctate and setose, punctures slightly larger and sparser anterolaterally; felt line broad, 0.4 × as long as T2 laterally. T3–5 densely punctate. Pygidium broad and slightly convex, lateral margins defined by distinct weakly bowed carina, posterior margin rounded and defined by indistinct carina, densely granulate throughout. S1 punctate, with weak darkened median longitudinal carina. S2 densely punctate. S3–5 densely punctate. S6 moderately punctate.

#### Material examined.

**Type material.** Holotype: ‘Brasil. mer\Sello.’ (green label partially handwritten) ‘6648’ ‘Type’ (red label) ‘Lectotypus\C.E. Mickel’ (red label partially handwritten) [1 female: ZMB]. **Other material.** 92 males and 101 females as follows: ARGENTINA: Entre Ríos: XII.1996 (Liebig, Zelich) [4 females, 4 males: AMNH]; Missiones: Dos de Mayo, XII.1989 (Foerster) [1 male: AMNH]; Puerto Esperanza, XII.1976 (M.A. Fritz) [3 males: AMNH]; XII.1973 (M.A. Fritz) [19 males: AMNH]; BRAZIL: Minas Gerais: Belo Horizonte, Museu de História Natural, 22.III.1998 (G.A.R. Melo) [1 female: DZUP]; 9 km E de Catas Altas, Serra da Caraça, 12.I.2012 (G.A.R. Melo) [1 female: DZUP]; 8 km S de Ouro Preto, Lago do Custódio, 20.I.2012 (G.A.R. Melo) [3 females: DZUP]; Serra do Caraca, S. Barbara, I.1970 (F.M. Oliveira) [1 male: EMUS]; Viçosa, M. do Paraiso, 5.I.1995 (G.A.R. Melo) [1 female: DZUP]; same city, XII.1944 (Wygodzinsky) [1 female: MNRJ]; Paraná: Capitão Leônidas Marques, Salto Caxias, 6–13.X.2004 (Soares, E.D.G.) [3 males: DZUP]; same locality, 13-20.X.2004 (Soares, E.D.G.) [5 males: DZUP]; same locality, 3-10.XI.2004 (Soares, E.D.G.) [12 males: DZUP]; Fazenda do Jordão, Posto Florestal, Salto Segredo, 6–10.X.2004 (Soares, E.D.G.) [1 male: DZUP]; Jaguatirica, Rio Capivari, 1.III.2003 (G.A.R. Melo) [1 male: DZUP]; Piraquara, Mananciais da Serra, 13.XII.2002 (Garcia, E.Q.) [2 males: DZUP]; same locality, 13.XII.2002 (Garcia, E.Q.) [1 female: DZUP]; same locality, 23.I.2003 (Garcia, E.Q.) [1 female: DZUP]; same locality, 2.II.2003 (Garcia, E.Q.) [1 female: DZUP]; same locality, 5.II.2003 (Garcia, E.Q.) [1 female: DZUP]; same locality, 8.II.2003 (Garcia, E.Q.) [1 female: DZUP]; same locality, 19.II.2003 (Garcia, E.Q.) [1 female: DZUP]; same locality, 26.II.2003 (Garcia, E.Q.) [3 females: DZUP]; same locality, 27.II.2003 (Garcia, E.Q.) [2 females: DZUP]; same locality, 14.III.2003 (Garcia, E.Q.) [3 females: DZUP]; same locality, 3.IV.2003 (Garcia, E.Q.) [1 female: DZUP]; same locality, 27.I.2001 (G.A.R. Melo) [5 males: DZUP]; Ponta Grossa, Parque Estadual da Vila Velha, 5.IV.2013 (K.A. Williams) [12 females: DZUP]; same locality, 6.IV.2013 (K.A. Williams) [10 females: DZUP]; same locality, 11.XII.2000 (Ganho & Marinoni) [1 female: DZUP]; same locality, 25.XII.2000 (Ganho & Marinoni) [1 female: DZUP]; same locality, 11.III.2002 (Ganho & Marinoni) [1 female: DZUP]; Prudentópolis, 8.II.1970 (J.S. Moure) [1 female: DZUP]; Tibagi, XII.1952 (Justus) [1 female: DZUP]; Rio de Janeiro: Itatiaia: Faz. Penedo, II.1943 (Wygodzinsky) [1 female: MNRJ]; same city, 700m, XII.1954 (W. Zikan) [1 female: MNRJ]; Petropolis, IV.1952 (C. Novais) [1 female: MNRJ]; Rio Grande do Sul: Arroio Grande, 101m, 32°13'S, 53°12'W, 9.IV.2004 (R.F. Kruger) [1 male: UFES]; Santa Catarina: Criciúma, Campus da UNESC, 16.XI.2002 (G.A.R. Melo) [1 male: DZUP]; Nova Teutonia, XI.1968 (F. Plaumann) [1 female: EMUS]; same locality, various dates (F. Plaumann) [16 females: MNRJ]; same locality, various dates (F. Plaumann) [3 females: YPM); same locality, XI.1968 (F. Plaumann) [2 males: YPM]; same locality, XII.1968 (F. Plaumann) [1 male: YPM]; same locality, XI.1980 (F. Plaumann) [1 female: DZUP]; São Paulo: Barueri, various dates (K. Lenko) [3 females: CASC]; same locality, V.1958 (K. Lenko) [1 female: MNRJ]; Batatais, I.1946 (J.S. Moure) [1 female: DZUP]; Cajuru, Fazenda Rio Grande, 2–18.XI.1999 (G.A.R. Melo & Nascimento) [2 males: DZUP]; same locality, 17.XI-2.XII.1999 (G.A.R. Melo & Nascimento) [3 males: DZUP]; same locality, 18.XII.1999–10.I.2000 (G.A.R. Melo & Nascimento) [3 males: DZUP]; same locality, 10.I-1.II.2000 (G.A.R. Melo & Nascimento) [4 males: DZUP]; Caraguatatuba, 1.III.1967 (M.E. Irwin) [1 female: CASC]; Cosmópolis, 22.I.1974 (J.G. Rozen et al.) [3 females: EMUS]; Est. Biol. Boracéia, 850m, 27.II.1967 (M.E. Irwin) [2 females: CASC]; Estação Ecológica de Jatai, Luís Antônio, 22.IV.1999 (G.A.R. Melo) [1 female: DZUP]; same locality, 16.X.1999 (G.A.R. Melo) [3 females: DZUP]; Riberão Grande, P.E. Intervales: ponto 2, 24°16'29"S, 48°25'17"W, 22.I.2010 (Perioto, N.W.) [2 males: MZSP]; same locality, 22.II.2010 (Perioto, N.W.) [1 male: MZSP]; same locality, 22.XII.2010 (Perioto, N.W.) [3 males: MZSP]; same locality, ponto 5, 24°16'23"S, 48°25'22"W, 22.I.2010 (Perioto, N.W.) [2 males: MZSP]; same locality, 22.IV.2010 (Perioto, N.W.) [2 males: MZSP]; same locality, 22.XII.2010 (Perioto, N.W.) [2 males: MZSP]; same locality, 22.XI.2010 (Perioto, N.W.) [1 male: MZSP]; Parque Ainhanguera, 28.II.1986 (C. Costa) [1 female: MZSP]; São Jose dos Campos, XII.1934 (H.S. Lopes) [1 female: MNRJ]; same locality, 5–20.V.1999 (E.R. DePaula) [1 male: EMUS]; Teodoro Sampaio, P.E. Morro do Diabo, Estrada do Angelim, 16.II.1999 (G.A.R. Melo) [1 female: DZUP]; PARAGUAY: Alto Parana: Estancia Dimas, 25°33'S, 55°13'W, 28–30.XI.2005 (U. Dreschel) [1 female: EMUS]; Canindeyú: Tava Yopoi, 24°22'S, 55°53'W, 26.X-4.XI.2007 (U. Dreschel) [1 female: EMUS]; Paraguarí: La Rosada, 28–30.IV.2006 (U. Dreschel) [1 female: EMUS]; San Pedro: Rio Ypane, Cororo, various dates (Fritz) [2 females, 2 males: AMNH, EMUS]; URUGUAY, Río Negro: Arroyo Negro, 15 km S Paysandu, 27–31.XII.1962 (R.G. Van Gelder) [3 males: AMNH].

#### Distribution.

This species apparently occurs throughout the forest and grassland regions of southern South America: Argentina, Brazil, Paraguay and Uruguay.

#### Host.

Unknown.

#### Remarks.

*Darditilla debilis* is a widely distributed and common species. Over 200 additional specimens were examined in MZSP and others were studied from various North American collections.

Males are associated with *Darditilla debilis* based on morphological similarity to *Darditilla bejaranoi* males and overlapping distribution with the females of *Darditilla debilis*, including five localities where both sexes were collected.

### Darditilla felina

Taxon classificationAnimaliaHymenopteraMutillidae

(Burmeister, 1854)

Figs 7 –8 , 21 –24

Mutilla felina Burmeister, 1854. Abh. naturf. Ges. Halle 2: 26. Holotype female, Brazil, Novo-Friburgo (MLUH, not examined).
Mutilla parasitica Smith, 1879: 213. Holotype female, Brazil, Constancia (NHM, examined), synonymized by Mickel (1964: 166).
Mutilla decorosa Kohl, 1882. Holotype female, Brasilia (NMW, examined through photographs). **syn. n.**
Mutilla decorosa Kohl: Nonveiller 1990: 113, ♀ (*incertae sedis*).

#### Diagnosis.

**Figure 7.**
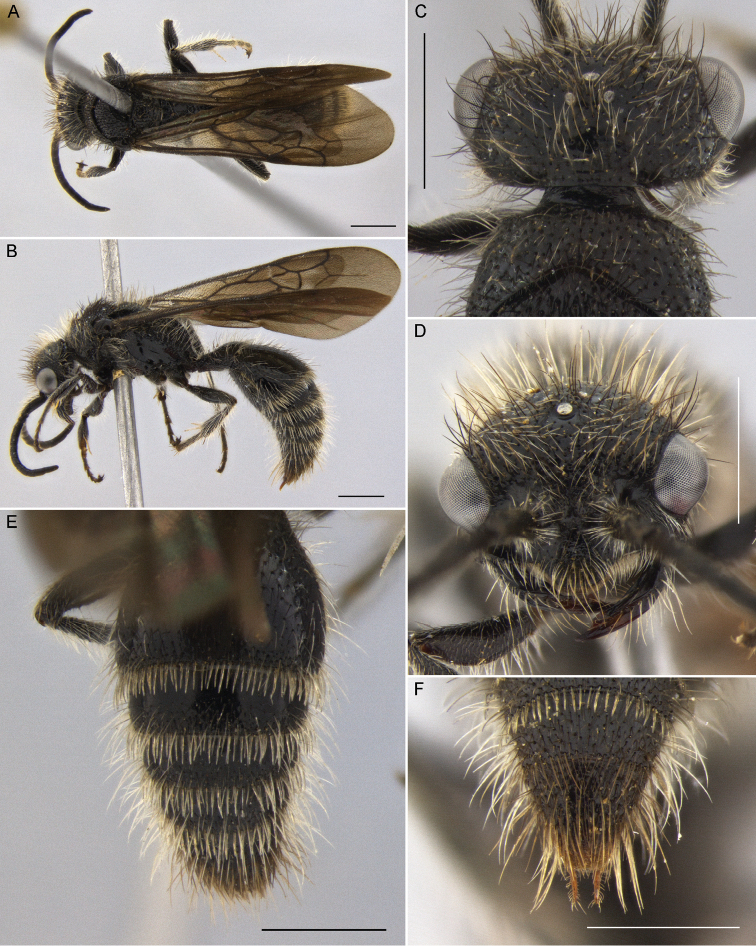
*Darditilla felina* (Burmeister), male: **A** habitus, dorsal view **B** habitus, lateral view **C** head, dorsal view **D** head, anterior view **E** metasoma, dorsal view **F** T5, T6 and pydigium, dorsal view. Scale bars: 1 mm.

**Male.** The male of *Darditilla felina* is easily recognized by having a unique clypeus, with the ventral margin, often hyaline, raised broadly lamellate (Fig. 7D), and the penis valve, which is unidentade apically (Fig. 23).

**Figure 8.**
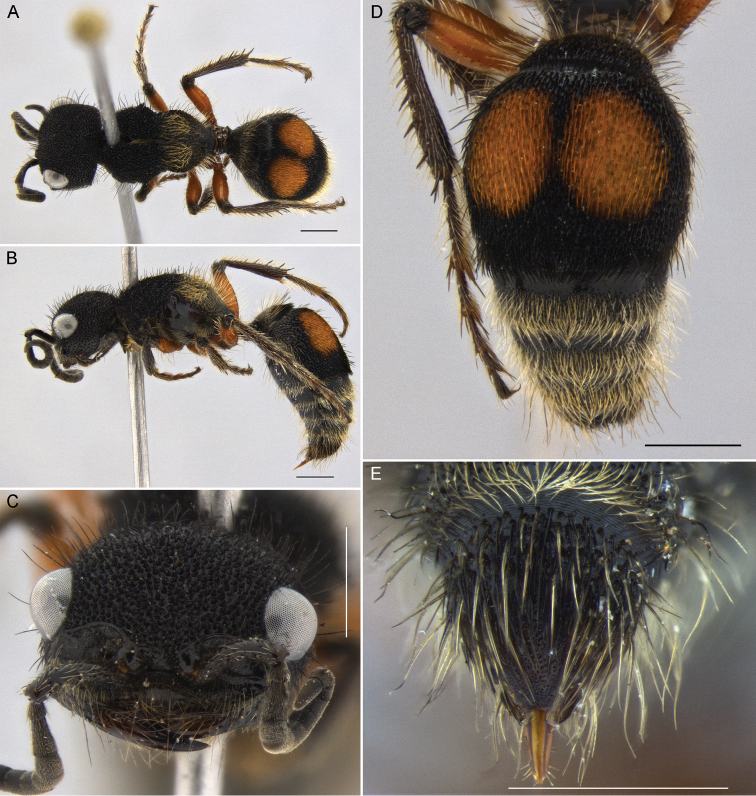
*Darditilla felina* (Burmeister), female: **A** habitus, dorsal view **B** habitus, lateral view **C** head, anterior view **D** metasoma, dorsal view **E** T5 and pydigium, dorsal view. Scale bars: 1 mm.

**Female.** This female can be separated from all other southern and southeastern Brazillian *Darditilla* by the large, coalescing lateral orange spots of T2 (Fig. 8D), and the large coalescing lateral patches of silver to pale golden setae on the propodeum (Fig. 8A).

#### Description.

**Male.** Body length 5.8–9.4 mm. *Coloration*. Body and appendages black, except mandibles and metasoma dark brown apically and ventral margin of clypeus often hyaline. Tibial spurs white. Forewing slightly and uniformly infuscated between veins, veins brown; hindwing slightly infuscated. Body clothed with whitish setae, except dorsoposterior portion of pronotum, axilla, scutellum, disc of T2, T6 and T7 with scattered brownish setae, mesoscutum and tegula with dark brown setae, bristles of tergal fringes silver to pale yellowish. *Head*. Rounded posteriorly. Head width 1.2 × pronotal width. Eye transversely ovate. Ocelli small; ocellocular distance 7.1 × length of lateral ocellus, interocellar distance 2.6 × lateral ocellus length. Occipital carina distinct, extending ventrally almost to level of oral fossa. Punctation on front dense, interspaces micropunctate; gena densely punctate, interspaces with sparse micropunctures; and vertex moderately punctate, interspaces glabrous. Gena ecarinate. Antennal scrobe broadly concave to eye margin, lacking dorsal carina or tubercle. Clypeus densely punctate, with small median concavity and raised broadly bilobate lamellate ventral margin. Scape unicarinate ventrally. Flagellomere 1 1.3 × pedicel length; flagellomere 2 1.6 × pedicel length. Mandible convergent to bidentate apex, dorsal carina gradually becoming obsolete near inner tooth; edentate ventrally. *Mesosoma*. Epaulets weakly produced. Pronotal dorsum densely punctate; anterior face obscurely punctate laterally and smooth medially; lateral face densely punctate. Tegula evenly convex, glabrous except with long recumbent setae anterolaterally and posteromesally. Mesoscutum with dense coarse punctures; posterolateral corner forming a small angulate lobe. Scutellum slightly convex, with coarse punctures. Axilla flat and punctate, except lateral margin with narrow vertical lamella. Metanotum surface obscured by dense shaggy mesally facing recumbent setae. Propodeum convex, broadly and deeply reticulate except smooth and shining adjacent to metapleuron. Mesopleuron moderately punctate, interspaces micropunctate. Metapleuron smooth and shining ventrally, setose and micropunctate dorsally. *Wings*. Forewing with moderate elongate sclerotized pterostigma; marginal cell broadly rounded; three submarginal cells, third scarcely delimited by obscure venation. *Legs*. Mid- and hind tibiae lacking strong spines, distinct apical secretory pore on inner surface near base of inner spur; spurs finely serrate on margins. *Metasoma*. T1gradually broadened from base, not constricted apically, sessile with T2, 0.5 × width of T2, sparsely coarsely punctate; apex with fringe of simple setae or thickened bristles. T2 with coarse to moderate punctures, interspaces smooth and generally broader than punctures; apex with fringe interspersed recumbent thickened parallel bristles; felt line 0.5 × lateral length of T2. T3–5 densely and finely punctate, covered with interspersed erect and recumbent setae, except fringes with row of bristles as in T2. T6 densely punctate, covered with less dense recumbent and erect setae. T7 in basal half with densely and coarsely punctures; posterior half forming oval pygidium margined laterally by a strong sharp carina, apical margin rounded, microgranulate with numerous large irregular rugae. S1 punctate and setose, with low medial longitudinal carina extending from base to apex. S2 moderately punctate. S3–6 moderately and finely punctate, with fairly sparse erect and recumbent setae. Lateral margins of S2–5 sometimes with similar bristles to those of T2–6. S7 transversely rectangular. Hypopygium coarsely punctate, posterior margin straight with small medial emargination. *Genitalia* (Figs 21–24). Paramere tapering apically, moderately curved ventrally on basal half, scattered setae along inner and lateral margins. Cuspis angulate apically, extending ~0.3 × free length of paramere, with densely setose oblique ventral surface. Digitus laterally compressed and rounded apically, extending ~0.2 × free length of paramere, asetose. Penis valve asetose, unidentate apically, basoventral margin with two minute teeth, basodorsal margin with slight tubercle.

**Figures 9–24.**
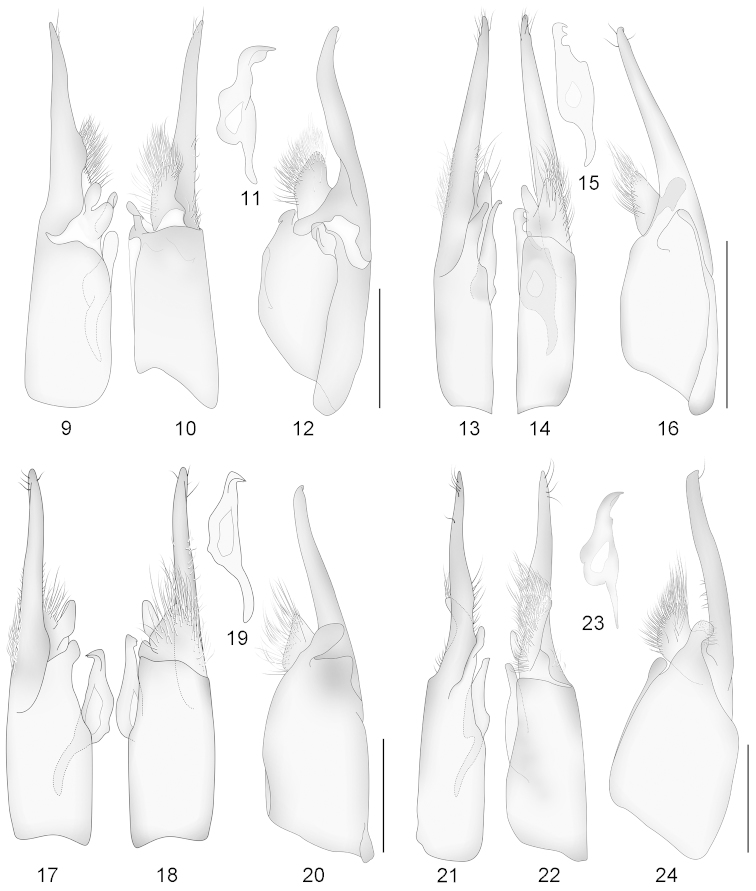
Male genitalia of *Darditilla* species: dorsal view (left), ventral view (middle), lateral view of penis valve (above), and lateral view with penis valve removed (right). **9–12** *Darditilla amabilis* **13–16** *Darditilla bejaranoi* **17–20** *Darditilla debilis* **21–24** *Darditilla felina*. Scale bars: 0.5 mm.

**Extended female diagnosis.** Body length 5.7–11.9 mm. *Coloration.* Body entirely black, except T2 with large, coalescing lateral orange spots and S2 often orange basomedially. Appendages variable, ranging from entirely black to entirely orangish. Tibial spurs whitish. Head with sparse erect black setae. Mesosoma with sparse erect black setae, except propodeum with coalescing large lateral patch of silver to pale golden setae. Posterior fringes of T1 and T2 black; T2 setae black anteriorly and posteriorly, pale golden laterally, and reddish orange on orange integumental spots; T3–6 clothed with silver to golden setae. *Head*. Transverse, posterior margin flat, occipital carina obscure. Head width 1.5 × pronotal width. Eye slightly ovate transversely, ommatidia distinct. Front, vertex and gena densely punctate. Genal carina well-defined, terminating in sharp angle posterior to hypostomal carina. Clypeus with transverse glabrous concavity, margined by dorsal and ventral carinae, between widely separated lateral teeth. Mandible tapering to apex, with tooth in basal third and in apical third, unarmed ventrally. Antennal scrobe with complete dorsal carina. Antennal tubercle punctate basally and on anterior face, glabrous dorsally. Scape simple, moderately punctate. Flagellomere 1 2.0 × pedicel length; flagellomere 2 1.25 × pedicel length. *Mesosoma*. Mesosomal length 1.4 × width. Mesosomal dorsum densely reticulate, propodeal reticulae broader. Lateral pronotal carina extending to epaulet, humeral angle obtusely angulate. Mesopleuron densely punctate and setose, posterior margin defined by vertical carina. Metapleuron and lateral face of propodeum smooth and shining dorsally with isolated fine setae, micropunctate and densely setose ventrally. In dorsal view, mesosoma broadened to anterior third, strongly narrowed at propodeal spiracle, propodeum abruptly broadened. Scutellar scale lacking. Propodeum convex, dorsal and lateral faces not obviously differentiated. *Legs*. Foreleg with a few long strong articulated spines on posterior/lateral margins of tarsomeres. Mid- and hind tibiae each with one rows of prominent spines, 5 spines in each row; apical spurs finely serrated laterally. Hind tibia with distinct secretory pore on inner/posterior surface near base of inner spur. *Metasoma*. T1gradually broadened from base, not constricted apically, sessile with T2, 0.5 × as wide as T2; anterior face moderately punctate and setose. T2 densely punctate, punctures slightly larger and sparser anterolaterally on orange spots; felt line broad, 0.6 × as long as T2 laterally. T3–5 densely punctate. Pygidium broad and slightly convex, lateral margins defined by distinct weakly bowed carina, posterior margin rounded and defined by indistinct carina, granulate. S1 punctate, with obscure blackish median carina. S2 moderately punctate. S3–6 densely punctate.

#### Material examined.

**Type material.** Holotypes: *Mutilla parasitica*, ‘CONSTANCIA\Jan 1857.\H.Clark’ ‘Mutilla\parasitica\ (Type) Sm’ (handwritten label) ‘Type’ (round, red-edged label) ‘B.M. TYPE\HYM.\15.871’ [1 female: NHM]; *Mutilla decorosa*, ‘Wthm.’ ‘6648’ ‘Brasilia\Macalú’ (handwritten label) ‘decorosa Kohl\Type’ (handwritten label) ‘decorosa\Type. Kohl’ (handwritten label) ‘HT decorosa’ (handwritten label) ‘Pseudomethoca\decorosa (Kohl)\det.R.Cambra 2012’ (handwritten label) [1 female: NMW]. **Other material.** 143 males and 36 females as follows: BRAZIL: Mato Grosso do Sul: Aquidauana, malaise 09, 20°26'07"S, 55°39'33"W, 11-26.X.2011 (Lamas & Nihei) [2 males: MZSP]; Paraná: Capitão Leônidas Marques, Salto Caxias, 3-10.XI.2004 (E.D.G. Soares) [18 males: DZUP]; Fazenda do Jordão, Posto Florestal, Salto Segredo, 6-10.X.2004 (E.D.G. Soares) [5 males: DZUP]; same locality, 13-20.X.2004 (E.D.G. Soares) [3 males: DZUP]; Piraquara, Mananciais da Serra, 2.XII.2005 (L.C. Rocha-Filho) [1 female: DZUP]; same locality, 2.I.2006 (L.C. Rocha-Filho) [1 female: DZUP]; same locality, 30.XI.2005 (L.C. Rocha-Filho) [2 males: DZUP]; same locality, 10.I.2001 (G.A.R. Melo) [2 females: DZUP]; same locality, 27.I.2001 (G.A.R. Melo) [1 male: DZUP]; same locality, 9.III.2002 (Garcia, E.Q.) [1 female: DZUP]; same locality, 24.IV.2002 (Garcia, E.Q.) [1 female: DZUP]; same locality, 12.XII.2002 (Garcia, E.Q.) [1 female: DZUP]; same locality, 5.II.2003 (Garcia, E.Q.) [2 females: DZUP]; same locality, 1.II.2003 (Garcia, E.Q.) [1 female: DZUP]; same locality, 8.II.2003 (Garcia, E.Q.) [1 female: DZUP]; same locality, 26.II.2003 (Garcia, E.Q.) [2 females: DZUP]; same locality, 27.II.2003 (Garcia, E.Q.) [2 females: DZUP]; same locality, 14.III.2003 (Garcia, E.Q.) [1 female: DZUP]; Ponta Grossa, Lageado, II.1957 (Justus) [1 female: DZUP]; Parque Estadual Vila Velha, 25.X.1999 (Ganho & Marinoni) [3 males: DZUP]; same locality, 1.XI.1999 (Ganho & Marinoni) [11 males: DZUP]; same locality, 8.XI.1999 (Ganho & Marinoni) [7 males: DZUP]; same locality, 15.XI.1999 (Ganho & Marinoni) [1 female, 6 males: DZUP]; same locality, 22.XI.1999 (Ganho & Marinoni) [10 males: DZUP]; same locality, 29.XI.1999 (Ganho & Marinoni) [2 males: DZUP]; same locality, 6.XII.1999 (Ganho & Marinoni) [2 males: DZUP]; same locality, 20.XII.1999 (Ganho & Marinoni) [1 female, 1 male: DZUP]; same locality, 27.XII.1999 (Ganho & Marinoni) [1 male: DZUP]; same locality, 23.X.2000 (Ganho & Marinoni) [4 males: DZUP]; same locality, 30.X.2000 (Ganho & Marinoni) [1 male: DZUP]; same locality, 6.XI.2000 (Ganho & Marinoni) [3 males: DZUP]; same locality, 13.XI.2000 (Ganho & Marinoni) [4 males: DZUP]; same locality, 20.XI.2000 (Ganho & Marinoni) [1 male: DZUP]; same locality, 27.XI.2000 (Ganho & Marinoni) [4 males: DZUP]; same locality, 4.XII.2000 (Ganho & Marinoni) [1 female, 3 males: DZUP]; same locality, 1.I.2001 (Ganho & Marinoni) [1 female: DZUP]; same locality, 29.X.2001 (Ganho & Marinoni) [1 female: DZUP]; same locality, 29.X.2001 (Ganho & Marinoni) [4 males: DZUP]; same locality, 5.XI.2001 (Ganho & Marinoni) [1 female: DZUP]; same locality, 12.XI.2001 (Ganho & Marinoni) [6 males: DZUP]; same locality, 26.XI.2001 (Ganho & Marinoni) [11 males: DZUP]; same locality, 3.XII.2001 (Ganho & Marinoni) [2 males: DZUP]; same locality, 26.XI.2001 (Ganho & Marinoni) [2 females: DZUP]; same locality, 24.XII.2001 (Ganho & Marinoni) [1 female: DZUP]; same locality, 28.I.2002 (Ganho & Marinoni) [1 female: DZUP]; same locality, 18.III.2002 (Ganho & Marinoni) [1 female: DZUP]; São José dos Pinhais, 17-27.XII.1984 (C.I.I.F.) [1 male: DZUP]; same locality, 22-29.X.1984 (C.I.I.F.) [1 male: DZUP]; same locality, 17-27.XII.1984 (C.I.I.F.) [1 male: DZUP]; Rio de Janeiro: Itatiala, 6 km NW de Itatiala, PN Itatiaia, 28.X.2011 (D.R. Luz) [1 female, 1 pair *in copula*: DZUP]; Novo Friburgo, I.2013 (P.C. Grossi) [1 male: DZUP]; Represa Rio Grande, 20.V.1967 (F.M. Oliveira) [1 female: DZUP]; Teresopolis, P.N. Serra das Orgãos, 22°26'S, 42°56'W, 31.X-5.XI.2004 (A.L.B.G. Peronti) [1 male: UFES]; Santa Catarina: Corupa, I.1954 (A. Maller) [1 female: MNRJ]; same locality, II.1954 (A. Maller) [2 females: MNRJ]; São Paulo: Americo Brasiliense Clube Nautico, Cerrado, 25-29.IX.1999 (M.T. Tavares) [1 male: UFES]; Cajuru, Fazenda Santa Carlota, 17.XI–2.XII.1999 (G.A.R. Melo & Nascimento) [2 males: DZUP]; Campos do Jordão, XI.1957 (K. Lenko) [1 female: DZUP]; Eug. Lefevre, 1.XI.1937 (Travassos, Lopes e Oiticica) [1 female: MNRJ]; Riberão Grande, P.E. Intervales: ponto 2, 24°16'29"S, 48°25'17"W, 22.XII.2009 (N.W. Perioto) [3 males: MZSP]; same locality, ponto 3, 24°16'28"S, 48°25'19"W, 23.XI.2009 (N.W. Perioto) [6 males: MZSP]; same locality, 22.I.2010 (N.W. Perioto) [2 males: MZSP]; same locality, 22.XI.2010 (N.W. Perioto) [2 males: MZSP]; same locality, ponto 4, 24°16'29"S, 48°25'21"W, 20.XII.2010 (N.W. Perioto) [2 males: MZSP]; PARAGUAY: San Pedro: Rio Ypane, Cororo, XII.1983 (M.A. Fritz) [1 male: AMNH]; same locality, 24-27.XI.1983 (M. Wasbauer) [1 male: EMUS].

#### Distribution.

This species is distributed throughout the Atlantic Rainforest of Brazil and Paraguay.

#### Host.

Unknown.

#### Remarks.

The sexes of *Darditilla felina* were associated based on collection of a mating pair in the *Parque Nacional do Itatiaia* in Rio de Janeiro state. DRL discovered the male and female together, already *in copula*, on leaf litter in a trail through the forest. The male that Burmeister (1854) originally associated with *Darditilla felina* was recognized as *Ephuta inaurata* (Smith, 1855) by Mickel (1964).

Both sexes of *Darditilla felina* have been examined from throughout the Atlantic Rainforest. The subspecies, *Darditilla felina agatas* Casal, 1968 differs from typical females of *Darditilla felina* in setal coloration and is known from two specimes from the Chaco region of Bolivia. It is unclear whether this is a valid species, valid subspecies, or a synonym of *Darditilla felina* without study of further specimens.

Females that key (Casal 1968a) to *Darditilla felina* have been examined from Rondônia. Although these females match *Darditilla felina* in coloration, their genal carina is different. *Darditilla felina* has the genal carina extending from the posterior head margin to below the eye, where it terminates at an angle; this putative new species has the genal carina extending nearly to the hypostomal carina where it gradually terminates. We refrain from describing the species at this time because the focus of this manuscript is southern and southeastern Brazillian *Darditilla* and the male is yet unknown.

Females show extensive variation in coloration of the legs. Many specimens have the legs entirely orange, while other specimens, including the female from the mating pair, have the legs partially darkened or entirely black. In an unpublished key to female *Pseudomethoca* types, Mickel separated *Darditilla felina* from *Mutilla decorosa* (Kohl, 1882) on the basis of leg color. Photographs of the type of *Mutilla decorosa* were provided by Dominique Zimmermann (NMW) and it is a perfect match for the orange-legged form of *Darditilla felina*. As such, we place *Mutilla decorosa* as a junior synonym of *Darditilla felina*.

## Discussion

These are the first valid species-level sex associations in *Darditilla*. The sex associations presented here support Casal’s (1965, 1968a) initial genus-level associations. Because his male diagnosis, however, was based on a single species, it needed to be altered, as provided above. Seven additional South American genera are recognized from both sexes, but do not yet have any published species-level associations: *Calomutilla* Mickel, 1952 (Quintero and Cambra 1996, Quintero and Cambra 2001); *Limaytilla* Casal, 1964 (Brothers 2006); *Neomutilla* Reed, 1898 (Quintero and Cambra 2001); *Pertyella* Mickel, 1952 (Quintero and Cambra 1996); *Suareztilla* Casal, 1968 (Casal 1968b); *Tobantilla* Casal, 1965 (Williams et al. 2011); and *Vianatilla* Casal, 1962 (Quintero and Cambra 1996). Discovery of species-level sex associations in these genera will likely lead to broader diagnoses for the genera and can verify, or potentially invalidate, these genus-level associations.

The initial sex associations for this project came from a field observation of a mating pair (*Darditilla felina*) and from a mating pair in a museum (*Darditilla bejaranoi*). These rare events provided the data needed for further discoveries. The male morphology analyzed from the first two sex associations was compared to other male mutillids and allowed us to associate the sexes of two other species (*Darditilla amabilis* & *Darditilla debilis*). There remain 31 *Darditilla* species known only from females (Nonveiller 1990). Data presented here could be vital for recognizing the males. Instead of relying on rare events, further progress can be made in mutillid sex associations by systematic study of the distribution and morphology of museum specimens.

## Supplementary Material

XML Treatment for Darditilla

XML Treatment for Darditilla amabilis

XML Treatment for Darditilla bejaranoi

XML Treatment for Darditilla debilis

XML Treatment for Darditilla felina
